# Suitability of anthrax (*Bacillus anthracis*) in the Black Sea basin through the scope of distribution modelling

**DOI:** 10.1371/journal.pone.0303413

**Published:** 2024-11-07

**Authors:** Margarida Arede, Alberto Allepuz, Daniel Beltran-Alcrudo, Jordi Casal, Daniel Romero-Alvarez

**Affiliations:** 1 Department of Animal Health and Anatomy, Veterinary Faculty, Universitat Autònoma de Barcelona, Barcelona, Spain; 2 FAO, Regional Office for Europe and Central Asia, Budapest, Hungary; 3 Department of Ecology & Evolutionary Biology and Biodiversity Institute, University of Kansas, Lawrence, Kansas, United States of America; 4 Research Group of Emerging and Neglected Diseases, Ecoepidemiology and Biodiversity, Health Science Faculty, School of Biomedical Sciences, Universidad Internacional SEK (UISEK), Quito, Ecuador; Satyawati College, University of Delhi, INDIA

## Abstract

The Black Sea basin has a strategic geographic location bridging Asia and Europe and depends on traditional livestock practices for their local economies. Anthrax, a zoonotic bacterial disease caused by *Bacillus anthracis*, poses a significant global threat impacting public health, food security, pastoralist communities, and national economies. The disease is endemic or sporadic in the Black Sea basin, however, the study of its distribution has seldom been addressed, despite its burden and the presence of historical *B*. *anthracis* burial sites in the region. The viability of *B*. *anthracis* in a particular region is going to be influenced by multiple environmental factors, such as soil composition, climate, vegetation, and host abundance. For characterizing the potential distribution of *B*. *anthracis* in the Black Sea basin and assessing the potential for anthrax outbreaks, we applied an ecological niche modelling framework using the Maxent algorithm. This machine-learning algorithm models species distributions based on presence data and background information from a specified calibration region. We analyzed multiple variable combinations and proposed a novel approach for interpreting in-risk anthrax areas. Our findings underscored the importance of host abundance to the anthrax dynamics in the region. We identified anthrax-suitable areas spanning central and eastern Türkiye, Armenia, southern Georgia, southern Russia, Bulgaria, southern and eastern Romania, Hungary, Moldova, and southern Ukraine, which align with findings from previous global and regional studies on the potential suitability of anthrax. The insights gained from our research may help to develop targeted interventions, such as awareness and educational campaigns about anthrax, supervision of anthrax-infected carcasses disposal, and the promotion of livestock vaccination in high-risk areas. Additionally, these results can inform policies to mitigate the spread of anthrax in pastoralist communities in the Black Sea basin and foster collaboration between veterinary and public health entities on anthrax control.

## Introduction

Anthrax, a zoonotic bacterial disease, is caused by *Bacillus anthracis*, a spore-forming, Gram-positive, and rod-shaped bacterium [1]. While wild and domestic ungulates are the primary hosts of *B*. *anthracis*, it can also affect other mammals, including humans [2, 3]. Ruminants typically become infected through environmental exposure by ingesting the pathogen’s spores when grazing or browsing, and humans are usually infected through occupational exposure to infected animal carcasses or animal products [1].

Anthrax is present in all continents, causing high yearly mortality in domestic livestock and wild animals, along with high morbidity in humans [1, 4]. As a result, this disease threatens worldwide public health, food security, the livelihoods of pastoralist communities, and national economies [1]. *B*. *anthracis* is endemic in areas of Sub-Saharan Africa, central and southwestern Asia, Central and South America, and limited regions within the United States (US). In Europe, the disease is sporadic in animals, with a higher prevalence in southern Europe and links to historical foci in northern areas [2].

In Eurasia, the agricultural reform and the defunding of veterinary and public health services that followed the collapse of the Soviet Union in 1991, led to an increase of vaccine-preventable diseases such as anthrax [5–7]. As of 2023, anthrax remained endemic in Türkiye, Azerbaijan, Georgia, and Moldova, and it was reported sporadically in Bulgaria, Romania, Ukraine, Belarus [8], and the Russian Federation [9]. However, limited surveillance and disease awareness in many of these countries contribute to underreporting and gaps in understanding its geographic extent [10]. Adding to these challenges, the effects of climate change are raising concerns about anthrax spreading to new areas. For instance, in 2016, thawing permafrost in northern Russia is hypothesised to have released anthrax spores, causing an outbreak that affected over two thousand reindeer and resulted in human deaths [11].

As the environmental availability of spores is a hallmark of *B*. *anthracis* exposure to hosts, characterizing its ecological niche has been proposed as a way to understand its distribution [12]. The concept of the ecological niche was first introduced by Grinnell [13] as a “limited range of ecological variables that could maintain a population without immigration”. This concept was later developed by Hutchinson [14] as a quantifiable ecological area that determines species fitness and survivorship [15]. By studying the *B*. *anthracis* ecological niche, we aim to describe the environmental patterns that support anthrax spores’ survival which eventually leads to hosts’ exposure in the Black Sea basin [16, 17].

The black steppe soils covering part of our study region favour the viability of *B*. *anthracis* spores [16, 18], due to its richness in calcium (which, with its high cation exchange capacity, attracts *B*. *anthracis* spores), high organic carbon content (a key component of soil organic matter), and neutral to alkaline pH [16, 19]. Additionally, anthrax occurrences have been linked to soils with increased nitrogen levels and to vegetation growth influenced by carcass decomposition [20]; as well as to climatic conditions with relative humidity above 60% [21], temperatures exceeding 15.5°C [18], and dry summers followed by heavy rain, which can further concentrate anthrax spores in the soil [22].

Traditional ecological niche modelling (ENM) relies on abiotic predictors (e.g. climate) to characterize a species distribution and considers biotic interactions (e.g. host dynamics) to have negligent effects in modelling, a hypothesis called the Eltonian noise effect [23]. However, there is growing evidence that its inclusion can be crucial to describe broad-scale species distributions, especially when modelling a disease system [24]. In this study, we explored ENM approaches based on various combinations of predictor variables, incorporating only abiotic (climate, soil, and vegetation) or introducing a biotic predictor (ruminant abundance) to assess whether the inclusion of ruminant abundance improved model performance. Additionally, we proposed a novel approach to visualize and interpret Maxent algorithm outputs by leveraging uncertainty levels to further refine the output. This allows us to suggest high-risk areas of potential *B*. *anthracis* outbreaks in the Black Sea basin with higher accuracy, which can guide decision-makers to prioritize awareness campaigns, surveillance, and control activities.

## Methods

This study explores the potential suitability of anthrax in the Black Sea basin through distribution modelling, using anthrax occurrences in domestic animals, from nine countries of the region, namely: Armenia, Azerbaijan, Belarus, Bulgaria, Georgia, Moldova, Romania, Türkiye, and Ukraine.

### Occurrence data and geoprocessing

We curated a database of *B*. *anthracis* confirmed georeferenced occurrences causing disease in domestic animal species (i.e. cattle, sheep, goats, swine, and equine) that have been reported in the participating countries between 2006 and 2021 (hereafter anthrax occurrences). The data were procured internally by FAO, sourced directly by national experts, or available online. The consolidated database included international repositories, such as EMPRES-i and the World Animal Health Information System (WAHIS), regional sources, as the Animal Disease Information System (ADIS), and national databases from Moldova and Türkiye. Finally, it includes anthrax occurrences from Deka *et al*. 2022 [25] (Table 1 in S1 File).

**Fig 1:**
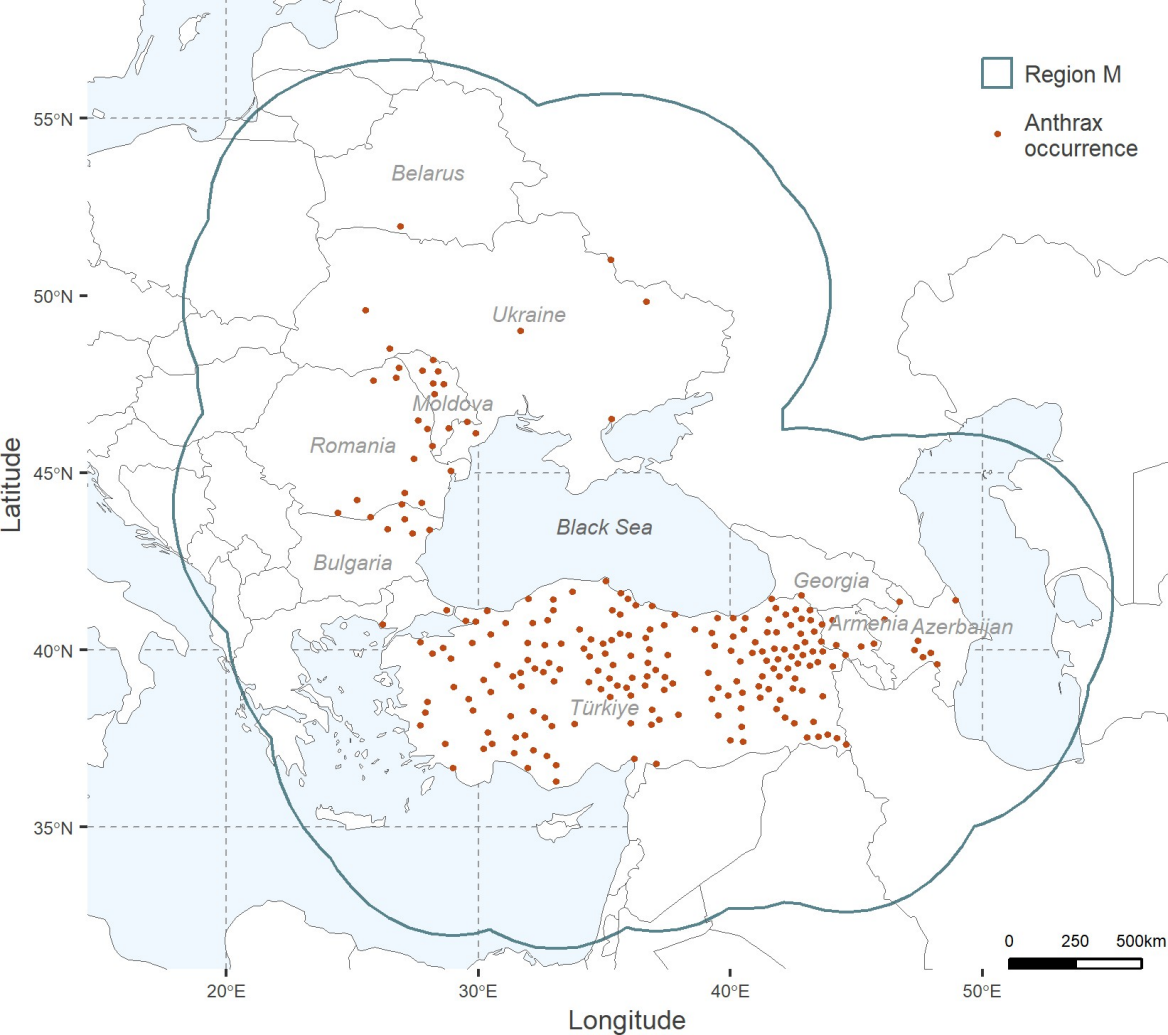
Anthrax georeferenced occurrences and calibration area (region M). *Bacillus anthracis* confirmed georeferenced occurrences (in dark orange) considered for the calculation of parameter M (outlined in teal). Maps were developed using shape files of the world from the public domain repository of Natural Earth (http://www.naturalearthdata.com/) and built using R Statistical Software (v4.2.1) [26].

Anthrax occurrence locations were processed in R Statistical Software (v4.2.1) [26]. We started by removing duplicates based on location and excluding records with a level of precision below three decimal degrees of latitude or longitude. Finally, to avoid overfitting due to spatial autocorrelation and sampling bias [25, 27], we applied a spatial thinning method of 30 km radius [28] using the R package *SpThin* [29]. This thinning threshold was selected based on findings from Romero-Alvarez *et al*. 2020, who assessed various thinning distances for *B*. *anthracis* occurrences at a continental level, and found that a 30 km spatial filter yielded a broader prediction with lower uncertainty [28]. The resulting thinned occurrences were used to develop ENMs, the final dataset comprised 226 occurrences (Fig 1).

### Calibration area

The calibration region, or parameter M, is the area used to calibrate the model. The correct delimitation of M is critical as it may impact any step of an ENM, from its parameterization, validation, and model comparison [30], to model outputs and interpretation [31, 32]. Parameter M should combine a spatial extent and environmental diversity that has been accessible to the studied species [33] during a time period that is relevant to the study [25, 30]. Here, we defined M by a buffer surrounding the occurrences which distance was calculated as the mean of the distances from each occurrence to the geographic centroid [34] (Fig 1).

### Variable selection

We selected four *B*. *anthracis* environmental predictors—climate (i.e. temperature and moisture), soil, and vegetation—and one host population variable. Variables were selected based on previous literature demonstrating the dependency of anthrax spatial distribution to these determinants [10, 28, 35].

We extracted 15 bioclimatic variables (i.e. seven temperature-related and eight moisture-related variables) from MERRAclim [36]. MERRAclim is a high-resolution global repository of satellite-based bioclimatic variables, offering advantages over other commonly used climate data sources for ENM, specifically, MERRAclim shows less uncertainty in interpolated values when compared with WorldClim [36]. Selected variables were included at a 5 arc-minute resolution for the period 2000 to 2010, partially aligning with the timeframe of our occurrences. In this study, we excluded the variables describing interactions between temperature and moisture—BIO8, BIO9, BIO18, and BIO19—due to known modelling artefacts [37–39]. Additionally, these variables were excluded because they combine domains that were analysed separately through principal component analysis (see below).

We selected four soil-related layers—pH, cation exchange capacity, carbon content, and nitrogen—due to their relevance for *B*. *anthracis* spores’ viability in soil. These variables were extracted from the Global Soil Information Facilities, SoilGrids, database [40], available at https://soilgrids.org/, at a 0-5cm depth and 250m resolution. SoilGrids is a repository for chemical and physical soil properties, based on a global compilation of soil profile data sets and environmental layers. It is the result of contributions from various national and international agencies and is developed by the International Soil Reference and Information Centre (ISRIC)—World Soil Information [40, 41].

As a measure of vegetation greenness, we used the Enhanced Vegetation Index (EVI) [42]. EVI’s version 6.1 was obtained through the 16-day composite images from the MOD13Q1 product at 250 m resolution [42] captured by the Moderate Resolution Imaging Spectroradiometer (MODIS) sensor, located in NASA’s TERRA satellite [43]. We processed satellite images to obtain the median from a composite of satellite images from 2005 to 2021 via Google Earth Engine [44]. EVI offers advantages over the Normalized Difference Vegetation Index (NDVI) in correcting atmospheric conditions and background noise [42].

Finally, we included a host population variable representing ruminant abundance, resulting from the sum of three raster layers for cattle, sheep, and goats abundance sourced from the Gridded Livestock World Distribution (GLW4) and adjusted to FAOSTAT 2015 country totals at 1 km resolution [45–48]. All variables were resampled to 1 km resolution using the *resample* function and bilinear method in R. Further details on anthrax environmental predictors and data sources are detailed Table 2 in S1 File.

To reduce high dimensionality and variable autocorrelation, we used a principal component analysis (PCA) [12, 49]. We used different sets of PCAs to determine three ENM approaches. For the first approach, we calculated principal components (PCs) for the entire set of 20 environmental variables. The two other approaches comprised PCs for each environmental domain (i.e. temperature, moisture, soil, and vegetation). The third approach treated environmental domains as in the second approach but also included the ruminant abundance variable. For each of these approaches, we used the PCs retaining at least 90% of the variation in the original data [50]. PCAs were developed using the *‘kuenm_rpca’* [51] function from *kuenm* package in R [51].

### Ecological niche modelling

Maximum Entropy algorithm (Maxent version 3.4.4) [52] was implemented to define ENMs. For this purpose, we applied the package *kuenm* [51] (https://github.com/marlonecobos/kuenm) in R Statistical Software (v4.2.1) [26] to calibrate Maxent ENMs and select optimal parameters for each of the three combinations of PCs as described earlier. Maxent is one of the most studied algorithms for ENM, as such, details on Maxent parameterization can be complex [51, 53]. For this study, we consider feature classes and regularization multipliers as parameters that might heavily influence the potential distribution of *B*. *anthracis* in the Black Sea Basin. Specifically, we investigated combinations of Maxent feature classes including linear, linear+quadratic, linear+quadratic+product as these forces the algorithm to recreate physiological plausible response curves [54, 55], and five regularization multipliers—i.e. 0.1, 0.5, 1, 1.5, and 2—as they determine a constrained or relaxed geographic prediction [56].

### Model evaluation

We partitioned anthrax occurrences randomly: 70% of occurrences for model training (calibration), and 30% of occurrences for model testing (evaluation) [57, 58]. Models were primarily evaluated and selected via the *kuenm* package [51] following a three-step approach. First, models were assessed for statistical significance (*p*-value<0.05) based on the partial area under the curve of the Receiver Operating Characteristic (*pROC*), a variation of the measure *ROC AUC* [59, 60]. To calculate this metric, we used, for each model, a threshold of 5% omission error (e.g. maximum excluded training occurrences) and 50 bootstraps. The *pROC* represents the ratio of correctly predicted occurrences to the area proportion with specified omission errors in the output models. *pROC* values greater than 1 (*p*-value < 0.05) indicate predictions that are better than random [59, 60]. Then, we selected those models with a lower omission rate (OR, threshold = 5%) [61]. The OR presents the model’s effectiveness in identifying true positive occurrences, taking into account those occurrences that were omitted (e.g. false negatives). Lastly, the resulting models were further narrowed down using the Akaike information criterion corrected for sample size (*AICc*) [62] to ensure low model complexity and good fit to the underlying data [58, 63, 64].

#### Final model

Final models were generated with the function *‘kuenm_mod’* from *kuenm* [51]. For the three modelling approaches, we specified the output format as logistic, with a continuous scale from 0 (non-suitable) to 1 (suitable). Additionally, we used 50 bootstrap replicates to calculate the median and assess model uncertainty, i.e. the difference between the rasters with maximum and minimum values. Final model outputs were categorized (i.e. suitable vs. non-suitable) considering the suitability value from the 95% of the calibration points (E = 5%) as threshold for binarizing the model [60].

From the three modelling approaches, we selected the best model based on the following criteria: lowest OR, lowest number of parameters, larger predicted area, and lowest uncertainty. Finally, to interpret the final model, we overlapped the best binarized model (i.e. suitable/unsuitable) with the uncertainty raster and considered highly suitable areas to those with less than the third quartile of uncertainty values.

## Results

A total of 1182 raw anthrax outbreak occurrences in domestic livestock, spanning from 2006 to 2021, were collated from various sources and used in the current study (Table 1 in S1 File). Cattle, sheep, and goats outbreaks accounted for 80.7%, 14%, and 4% respectively, representing the majority of studied outbreaks (98.7%). The remaining occurrences represented outbreaks attributed to horses and swine (1.3%). Over the studied period, the cumulative frequency of anthrax occurrences started increasing in July, peaked in September (n = 193) at three times the mean for the first six months of the year (n = 65), and gradually decreased until December (n = 62, S1 Fig).

Each of the three explored approaches resulted in 15 candidate models, reflecting combinations of three feature classes and five regularization multiplier values. The three best-fitting models were identified through the described three-step framework (Table 1).

**Table 1:** Parameters of ecological niche models categorized by principal component analysis (PCA) approach.

Approach	Selected features	Selected RM	No. Predicted pixels	pROC significance	OR-5%	AICc	No. of parameters
**APPROACH 1:**	LQP	0.1	34,917	<0.05	0.0294	4,732.26	20
ALL VARIABLES PCA							
**APPROACH 2:**	LQP	0.5	25,895	<0.05	0.0441	4,634.63	41
PCA BY DOMAIN (ENV ONLY)							
Approach 3:	**LQ**	**0.5**	**34,323**	**<0.05**	**0.0147**	**4,715.51**	**18**
**PCA by domain + host population variable**							

The best model for each approach was selected using a three-step selection framework (i.e. *pROC*, omission rates [OR], and *AICc*). *AICc*: Akaike information criterion corrected for sample; Features: L = linear, LQ = linear+quadratic, LQP = linear+quadratic+product; PCA: principal component analysis; *pROC*: partial area under the Receiver Operating Characteristic; OR: omission rate; RM: regularization multiplier.

**Fig 2:**
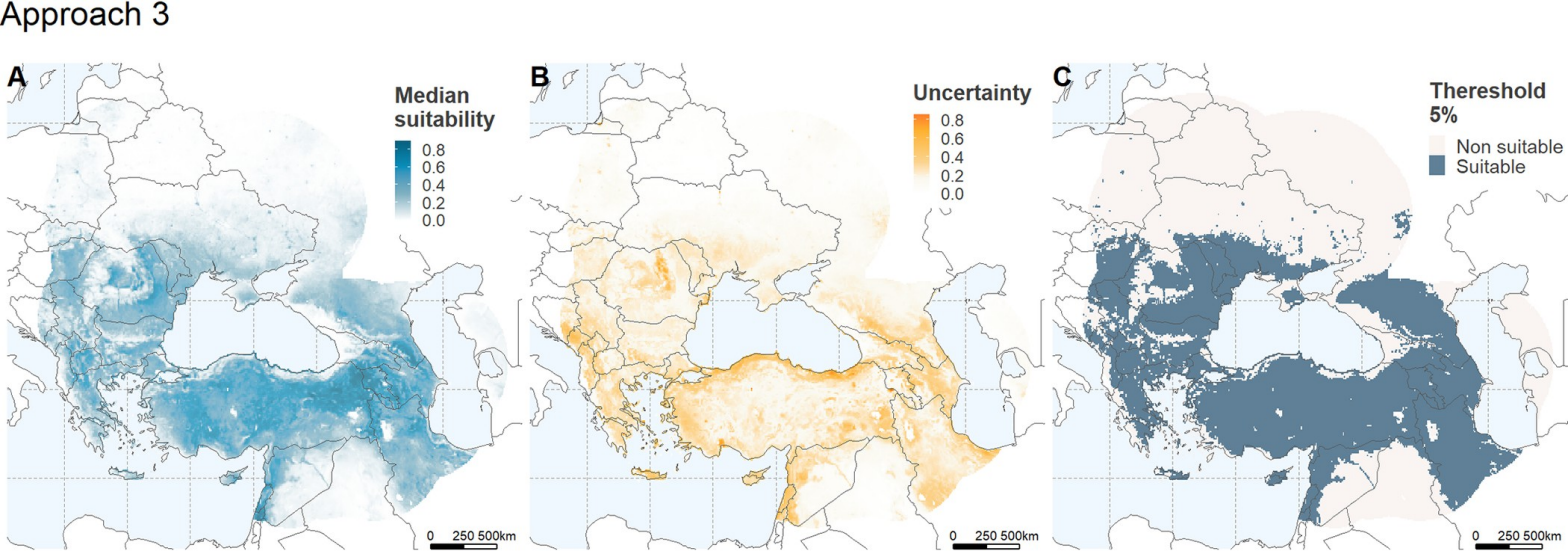
Ecological niche model outputs for *Bacillus anthracis* in the Black Sea basin. Model outputs for the selected best model for *B*. *anthracis* using principal components (PCs) by domain plus the host population variable based on ruminant abundance (i.e. approach 3; Table 1). Maps depict (A) continuous suitability, (B) uncertainty, and (C) a binary map of suitability using a 5% threshold. Maps were developed using shape files of the world from the public domain repository of Natural Earth (http://www.naturalearthdata.com/) and built using R Statistical Software (v4.2.1) [26].

The model output for *B*. *anthracis* developed using a PCA per environmental domain plus the variable representing ruminant abundance in the studied area were selected as the best overall model (i.e. approach 3; Table 1). This model yielded a wider prediction with lower uncertainty and presented a lower OR with a lower number of parameters than the two other approaches (Table 1). To generate this ENM approach, we retained the first three PCs for temperature and soil, explaining 98.83% and 95.77% of their respective domains, the first two PCs explaining 99.44% of the moisture domain, and one PC each for EVI and ruminant abundance. Models’ median, uncertainty, and areas suitable and non-suitable for *B*. *anthracis* at 5% threshold are illustrated in Fig 2. Outputs for the other two approaches can be found in the Fig 1 in S2 File. We highlight that the temperature and soil domains had the highest contribution to the final selected model accounting for 38.2 and 32.9%, whereas similar contributions were attributed to EVI and ruminant abundance, at 10.3% and 9.9%, respectively (Table 1 in S2 File).

**Fig 3:**
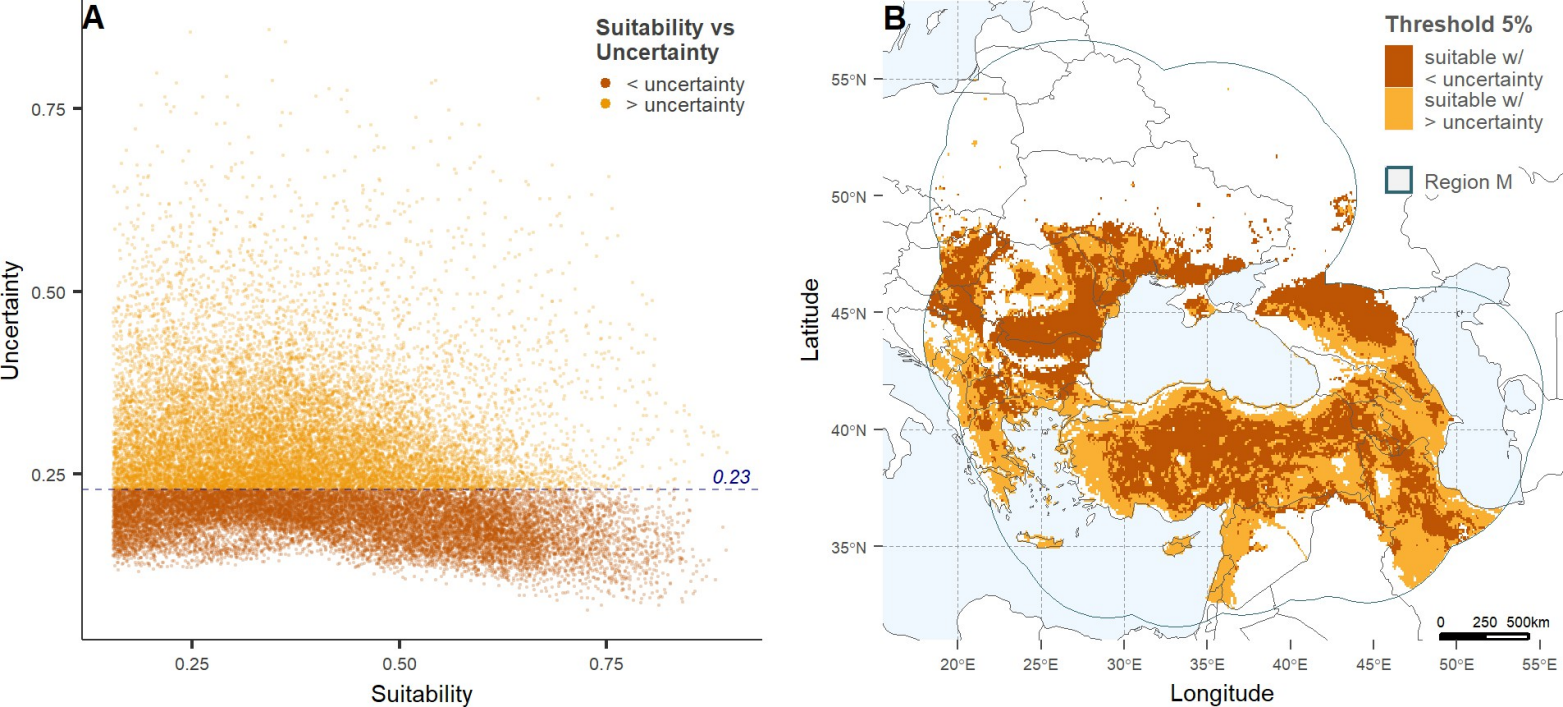
Suitability versus uncertainty regions for the best-selected model of the potential distribution of *Bacillus anthracis*. (A) Illustrates the correlation between continuous anthrax suitability and uncertainty for the best model (Table 1, Fig 2). High uncertainty was defined by a cut-off set as the third quartile across all uncertainty values (> = 0.23). (B) Depicts the 5% binary output of anthrax suitability with higher (orange) and lower (ochre) uncertainty. Maps were developed using shape files of the world from the public domain repository of Natural Earth (http://www.naturalearthdata.com/) and built using R Statistical Software (v4.2.*1)* [26].

We contrasted suitable areas for anthrax in the overall best model binary map with varying levels of model uncertainty. Low uncertainty was defined here as those pixels with values below the third quartile of the uncertainty range (i.e. Q3 = 0.23; Fig 3A). Regions identified as highly suitable with low uncertainty (Fig 3B) span western to central Armenia, extending into the southwest of Azerbaijan; they include a limited area in the northeast of Azerbaijan and the southern border region of the Russian Federation; the interior regions of the Islamic Republic of Iran and southern Russian Federation; as well as the interior eastern, central, and central-south areas of Türkiye (Fig 3B). Additionally, anthrax suitability is also observed in centre south and north Bulgaria and south and east Romania, centre east of North Macedonia, north of Serbia, southeast of Hungary, centre to south of Moldova, and the south coast of Ukraine with the Black Sea (Fig 3B).

Regions with high suitability and with low uncertainty where no anthrax occurrences have been reported (Figs 1 and 3B) can be found in the southern interior of the Russian Federation, the interior of the Islamic Republic of Iran, the central southern region of Bulgaria, central-east of North Macedonia, northern Serbia and centre to east of Hungary. Conversely, regions where anthrax cases have been reported, yet are depicted in our models as areas of low anthrax suitability, are primarily seen in central to northern regions of Ukraine and southern regions of Belarus. High suitability areas with high uncertainty are observed along the coast of southern Türkiye with the Black Sea, the west coastal area of Türkiye with the Mediterranean Sea, and the southern-east region of Türkiye along the border of the Republic of Iraq and the Islamic Republic of Iran.

## Discussion

Through the scope of distribution modelling, we found highly suitable regions for *B*. *anthracis* survival in the Black Sea basin; these areas might well benefit from investment and resource allocation for the control and prevention of anthrax outbreaks. Our model’s predictions agreed with findings from previous studies conducted at various geographical scales. Suitable areas identified for anthrax spanned from central to eastern Türkiye, Armenia, southern Georgia, the southern Russian Federation, Bulgaria, southern and eastern Romania, Hungary, Moldova, and southern Ukraine. These areas are similar to those found by recent studies exploring the ecological niche of *B*. *anthracis* at a global scale [10, 25], as well as a study specifically focused on northern latitudes [65]. Additionally, our model found anthrax-suitable areas with low uncertainty in northeast Azerbaijan, consistent with anthrax spatial clusters observed between 2000 and 2010 [6]; and the Odesa region in Ukraine, converging with a publication reporting *B*. *anthracis* in environmental samples and animal anthrax cases in this area [5]. Finally, we highlight that although our model did not include anthrax occurrences from Georgia, it accurately predicted the southeastern region of this country as suitable for anthrax, corroborating previous reports (Pers. Comm. T. Chaligava). However, it was unable to predict similar suitability in central to northern regions of Georgia, where both livestock (Pers. Comm. T. Chaligava) and human anthrax cases [66] have been documented.

There is a well-established spatio-temporal link between human and livestock anthrax cases due to the high occupational nature of anthrax in humans [1]. In this regard, our model corroborates the high incidence of human and livestock anthrax cases found in eastern provinces of Türkiye, clustering around animal trade centres and large international commercial roads [67, 68], and linked with livestock trade routes between eastern and western Türkiye and from the centre Anatolia to the southern and northern parts of the country [68].

In our assessment of Maxent ENMs with various variable combinations, we found that including ruminant abundance—i.e. biotic variable—improved model performance and was an important parameter in selecting the best overall model of anthrax suitability in this region. This finding emphasizes the importance of incorporating biotic variables in disease distribution models, highlighting the relevance of biotic interactions in disease systems [24]. Consistent with previous studies, which identified livestock abundance as a significant factor in anthrax distribution [19, 35, 65, 69–71], our findings further reinforce the role of ruminants as the most susceptible hosts to *B*. *anthracis*, playing a key role in the maintenance and transmission of anthrax [72].

In our study region, where ruminant production is a critical livestock subsector [73–82], our model identified suitable areas for anthrax that largely match rural settings where pastoralism is widely practiced [83]. This finding aligns with previous research linking anthrax outbreaks to rural, ’poor livestock-keeping’ communities and pasture contamination, as highlighted in a review on neglected and endemic zoonoses [84]. Furthermore, a case-control study in Georgia found that practices typically associated with commercial farming, such as keeping animals in covered areas or barns and vaccinating for anthrax within the last two years, were associated with a lower likelihood of disease occurrence [85]. Similarly, Carlson *et al* 2019 [10] suggested higher human anthrax risk in rural areas and observed increased human and livestock anthrax vulnerability in rainfed systems across arid and temperate landscapes in the same region (Eurasia).

Soils and temperature had the highest contribution percentage to our model (Table 1 in S2 File). Chernozem or black steppe-type soils, prevalent in eastern Europe [86] and partially covering our M region, are rich in organic matter and calcium with a pH above neutral. These soil characteristics create favourable conditions for anthrax sporulation and persistence [22] and have been associated with anthrax epidemics [16]. Additionally, our model identified the southern part of the M region, where the mean annual temperature is higher, as suitable for anthrax. This result aligns with established knowledge that anthrax viability increases in areas with temperatures exceeding 15 ⁰C [3] and is further supported by results from Carlson *et al*. 2019 and Walsh *et al*. 2018 [10, 65]. Furthermore, cumulative anthrax occurrences were higher between July and October, a period characterized by higher temperatures and drier conditions across the region [87], which facilitate the mechanical dispersion of anthrax spores [18]. Such temporal pattern has also been observed in Azerbaijan [88], Türkiye [89] and Kyrgyzstan [90]. During this time, ruminants graze in local pastures or migrate to summer pastures, and as the grass shortens, they graze closer to the soil, increasing their risk of exposure to *B*. *anthracis* spores [90]. Moreover, the high temperatures may induce nutritional stress in ruminants, compromising their immunocompetence and making them more susceptible to the disease [91].

Some of the few anthrax occurrences in the northern M region were missed by our final model (Fig 3). This discrepancy may be due to the low mean annual temperature at these latitudes, which theoretically hinders anthrax viability [3]. However, during the summer months, temperatures can still enable significant *B*. *anthracis* sporulation [3]. In contrast, Deka *et al*. 2022 [25] showed “very high” and “high” suitability for anthrax in parts of our northern region M, diverging from our findings. Additionally, anthrax cases in Ukraine and Belarus were reported sparingly, likely due to rigorous documentation and management of biothermal pits and infected burial grounds [5]. These areas are subject to strict legislation prohibiting construction, agricultural and pastoral practices without prior disinfection. The low number of cases in these countries may also be explained by the prevalence of intensive livestock production systems where ruminants are typically confined, and pastoral practices are uncommon, limiting their exposure to anthrax spores. Nevertheless, despite the current suboptimal environmental conditions for anthrax viability in these areas, climate change-led extreme weather events, such as warmer temperatures, high precipitation and droughts [92] are expected to increase anthrax risk in the future [25, 65].

Beyond environmental determinants, anthrax outbreaks have also been associated with a range of socio-economic factors including food security, disease awareness, cultural and religious practices, and access to veterinary services and healthcare. These factors are closely tied to livestock production practices, such as pastoralism, seasonal movements, disease surveillance capacity, vaccination use and coverage, and the implementation of biosecurity measures [85]. The emergence and reemergence of anthrax are particularly prevalent in regions affected by poverty [84]. For instance, eastern Anatolia in Türkiye, with a low gross domestic product per capita and rural population dependent on agriculture and livestock breeding, has a high incidence of anthrax, exacerbated by informal animal movements during religious festivities [67, 93]. Similarly, a case-control study in Georgia found that farmers affected by livestock anthrax cases had lower education and socioeconomic levels [94]. Further research on the impact of socioeconomic factors on anthrax risk in livestock and humans would complement our study and guide targeted interventions in the region.

Our regional-scale map illustrating anthrax suitability complements existing studies targeting this region at broader scales [10, 25, 65, 71]. In our study, we explicitly incorporated uncertainty measures into our final predictions, aiming to highlight and define more accurately potential anthrax suitable areas. The inclusion of uncertainty in the final outputs of ENMs is seldom implemented [10, 25, 28, 35], and we advocate for its consideration, especially in ENM studies exploring pathogens.

The modelling strategy applied in our experiment is comprehensive and follows state-of-the-art approaches for ENM in the study of infectious diseases. Specifically, we used automated scripts (i.e. *kuenm*) to explore various Maxent parameters, integrated uncertainty into model predictions, and tested multiple sets of models at once (Table 1, Fig 2, Fig 1 in S2 File). However, further refinements are still needed in ENM experiments, and therefore we disclose limitations related with occurrence data treatment and variable and algorithm selection [95]. In our study, occurrence data was clustered in specific regions, which may have contributed to model overfitting despite the implementation of occurrence thinning strategies [96]. For this study, we have used a spatial thinning approach using a ratio of 30 km based on previous studies [28]. However, evidence on best practices for occurrence thinning is controversial with experiments in favour and against of thinning strategies [96, 97]. Further research on this modelling step is granted and potentially will show that a rule-for-all to control for occurrence autocorrelation is difficult to attain. Additionally, while we included variables that directly contribute to *B*. *anthracis* survival, some might have been overlooked, as any model is ultimately an heuristic representation of reality [16]. Finally, regarding algorithm selection, the use of multiple algorithms and ensemble modelling as a definitive model remains an open discussion in the field [98]. For this study, we chose Maxent because it is one of the most comprehensively studied algorithms, and its parameterization and model selection strategies have proven effective in characterizing infectious diseases [12].

## Conclusions

Our study identified high-risk areas for anthrax across central and eastern Türkiye, Armenia, southern Georgia, southern Russia, Bulgaria, southern and eastern Romania, Hungary, Moldova, and southern Ukraine. These findings are critical for prioritizing resource allocation and implementing anthrax management interventions in the region.

Leveraging uncertainty levels and explicitly including them in our modelling approach improved the reliability of the potential suitable and non-suitable regions for anthrax identified in our final maps. We believe this approach also facilitates the interpretability of our results and enhances their utility for decision-makers and stakeholders.

The inclusion of ruminant abundance as a biotic variable in our modelling framework improved model performance, highlighting the importance of host-pathogen interactions in the study region.

Overall, anthrax poses a significant threat to ruminant production which is essential for the economies and subsistence of rural populations in the Black Sea region. We anticipate that the risk maps generated in this work offer comprehensive insights into anthrax distribution in this region, providing valuable guidance for targeted interventions to mitigate the impacts of this disease both at veterinary and public health levels.

## Supporting information

S1 Fig Seasonal trend of *Bacillus anthracis* georeferenced occurrences during our study period. (TIF)

S1 File Anthrax occurrences data sources, environmental domains, and R packages used in the current study. (PDF)

S2 File Description of model outputs for non-selected models and Maxent output for selected approach. (PDF)
