# Supplementary figures and images for: Suitability of anthrax (Bacillus anthracis) in the Black Sea basin through the scope of distribution modelling
Source: PLoS One. 2024 Nov 7;19(11):e0303413. doi: 10.1371/journal.pone.0303413 (PMC11542877; doi:10.1371/journal.pone.0303413)

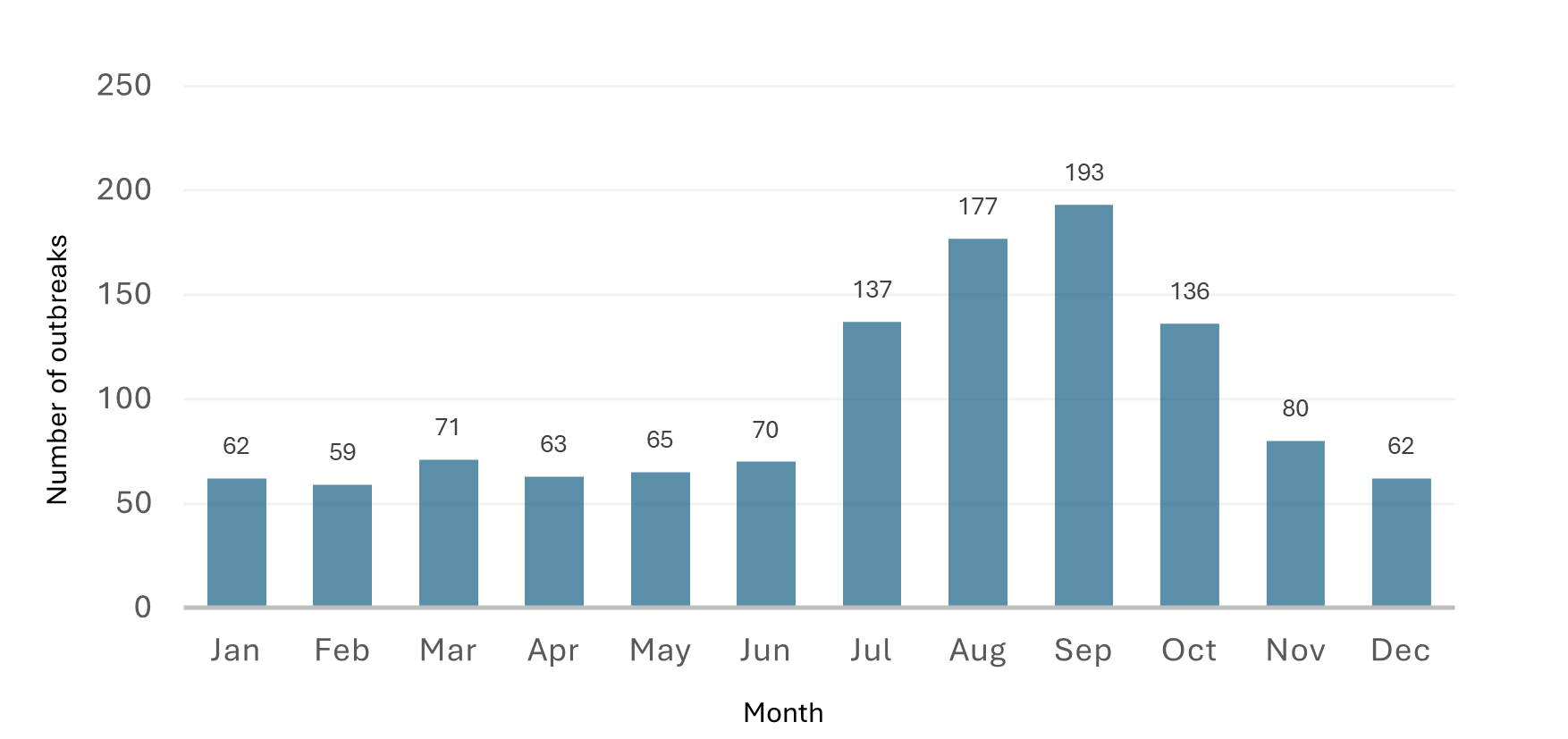

Supplement: S1 Fig — (TIF) [file pone.0303413.s001.tif]
