# Supplementary material for: Suitability of anthrax (Bacillus anthracis) in the Black Sea basin through the scope of distribution modelling
Source: PLoS One. 2024 Nov 7;19(11):e0303413. doi: 10.1371/journal.pone.0303413 (PMC11542877; doi:10.1371/journal.pone.0303413)
Supplement: S1 File — (PDF) [file pone.0303413.s002.pdf]

Suitability of Anthrax (*Bacillus anthracis*) in the Black Sea Basin through the scope of distribution modelling

Margarida Arede, Alberto Allepuz, Daniel Beltran-Alcrudo, Jordi Casal, Daniel Romero-Alvarez

S1 File: Anthrax occurrences data sources, environmental domains, and R packages used in the current study.

- **Data sources used to extract anthrax occurrences in the study.**
- **S1 File Table 1. Anthrax outbreaks in domestic animals (2006 to 2021).**
- **S1 File Table 2. Selected variable domains and respective sources.**
- **S1 File Table 3. Packages used in R programming language across this manuscript.**
- **S1 References**

Data sources used to extract anthrax occurrences in study.

Bacillus anthracis confirmed occurrences from 2006 to 2021 were compiled from various sources, including international and regional notification information systems, national datasets, and a scientific article. These databases are further detailed in the subsections below and S1, Table 1.

Global datasets

The Global Animal Disease Information System from the Food and Agriculture Organization (FAO), known as EMPRES-i+, is an online platform available at https://empres-i.apps.fao.org/diseases. It consolidates worldwide animal disease events information received from official and unofficial sources that are then verified and validated by FAO in coordination with the World Organisation for Animal Health (WOAH) and the World Health Organization (WHO). From this data source, our final dataset comprised a total of 52 anthrax outbreaks recorded between 2006 and 2021 [1].

The WAHIS (World Animal Health Information System) is the reference database of the WOAH. Anthrax cases for countries classified as free or sporadic are reported to WOAH as immediate notifications or follow-up reports and can be accessed through WAHIS Reports—animal disease events, which are available at https://wahis.woah.org/#/event-management. Anthrax outbreak locations from 2006 to 2021 for participating countries following these criteria include Armenia, Azerbaijan, Romania, and Ukraine [2].

Regional datasets*

The Animal Disease Information System (ADIS) is a disease management tool from the European Commission that was designed to document the evolution of the status of important infectious animal diseases (identified in the Animal Health Law (AHL)). This tool has the objective to ensure a rapid exchange of notifications between competent responsible authorities of EU member countries. Anthrax event notification data were obtained by FAO and was available for Bulgaria, Romania, Turkey, and Ukraine, a total of 563 outbreak points ranging from 2015 to 2021 were included in our original database [3].

National Datasets*

National datasets for georeferenced anthrax events were obtained from Moldova and Türkiye through national focal points. These data ranged from 2010 to 2016 for Moldova, and from2016 to 2021 to Türkiye.

Other

A subset for our study region and species of the global database for naturally occurring anthrax compiled by Deka et al. [4] was used to complete our database.

S1 File Table 1. Anthrax outbreaks in domestic animals (2006 to 2021). Number of outbreaks extracted from each data source.

	**Armenia**	**Azerbaijan**	**Belarus**	**Bulgaria**	**Moldova**	**Romania**	**Türkiye**	**Ukraine**	Total
**ADIS**	-	-	-	8	-	10	541	3	562
**EMPRES-i+**	9	15	1	-	1	16	1	8	51
**Focal point**	-	-	-	-	12	-	504	-	516
**WAHIS-events**	8	15	-	-	-	13	-	8	44
**Publication** [4]	2	2	-	2	-	2	-	1	9
**Total**	19	32	1	10	13	41	1046	20	1182

*These data were acquired under contractual agreements that require confidentiality concerning exact locations and any individual identifiable information. Access to this data may be granted upon request to the relevant entities, subject to adequate justification. The contacts of the relevant entities follow: Animal Disease Information System (ADIS) (link: https://food.ec.europa.eu/animals/animal-diseases/animal-disease-information-system-adis_en), Bulgaria (Central Office of the Bulgarian Food Safety Agency, bfsa@bfsa.bg), Romania (National Sanitary Veterinary and Food Safety Authority (A.N.S.V.S.A.), office@ansvsa.ro), Türkiye (Republic of Türkiye ministry of agriculture and forestry general directorate of food and control, tarimbilgi@tarim.gov.tr), Ukraine (State Service of Ukraine on Food safety and Consumer Protection (SSUFSCP), info@dpss.gov.ua), Moldova (National Food Safety Agency (ANSA), info@ansa.gov.md).

S1 File Table 2. Selected variable domains and respective sources. Details on resolution, source and availability of the four environmental domains: temperature, moisture, vegetation index, and soil; and one demographic variable: ruminant abundance, used in study.

Data domains & variable description	Variable short name	Spatial and temporal resolution	Source	Availability
**TEMPERATURE (9 layers) ***		**5 arc minutes / 2000-2010**	MERRAclim [5]	https://datadryad.org//resource/doi:10.5061/dryad.s2v81
Annual mean temperature	BIO1			
Mean diurnal range temperature	BIO2			
Isothermality	BIO3			
Temperature seasonality	BIO4			
Maximum temperature of the warmest month	BIO5			
Minimum temperature of the coldest month	BIO6			
Temperature annual range	BIO7			
Mean temperature of warmest quarter	BIO10			
Mean temperature of coldest quarter	BIO11			
**HUMIDITY/MOISTURE (6 layers) ***		**5 arc minutes / 2000-2010**	MERRAclim [5]	https://datadryad.org//resource/doi:10.5061/dryad.s2v81
Annual mean specific humidity	BIO12			
Specific humidity of most humid month	BIO13			
Specific humidity of least humid month	BIO14			
Specific humidity seasonality	BIO15			
Specific humidity mean of most humid quarter	BIO16			
Specific humidity mean of least humid quarter	BIO17			
**VEGETATION INDEX (299 layers)**		**250 m / 2005-2021**	Moderate Resolution Imaging Spectroradiometer (MODIS)[6]	https://lpdaac.usgs.gov/products/mod13q1v061/
Enhanced Vegetation Index MOD13Q1 product Version 6.1	EVI			
**SOILS (4 layers)**		**250 m / 2012-2016**	SoilGrids [7,8]	https://soilgrids.org/#!/?layer=TAXNWRB_250m&vector=1

Cation exchange capacity of soils at two depths	CECSOL†			
Soil organic carbon content at two depths	ORCDRA†			
Soil pH x 10 in H_2O at two depths	PHIHOX†			
Nitrogen	Nitrogen†			
**RUMINANTS' ABUNDANCE**** - Sum of raster layers for domestic ruminants' distribution GLW4		**5 arc minutes/ 2015**	Gridded Livestock of the world − 2015 (GLW4)[9]	
Global cattle distribution in 2015 (5 minutes of arc)	5_Ct_2015_Da.tif		[10]	https://dataverse.harvard.edu/file.xhtml?fileId=6769711&version=1.0
Global goats distribution in 2015 (5 minutes of arc)	5_Gt_2015_Da.tif		[11]	https://dataverse.harvard.edu/file.xhtml?fileId=6769696&version=1.0
Global sheep distribution in 2015 (5 minutes of arc)	5_Sh_2015_Da.tif		[12]	https://dataverse.harvard.edu/file.xhtml?fileId=6769626&version=1.0

*Bioclimatic variables combining information from temperature and humidity (BIO-8, BIO-9, BIO-18 and BIO-19) were not included in the analysis [13].

**Excluded global buffalo distribution.

†Depths considered for each variable: 0-5 cm.

S1 File Table 3: Packages used in R programming language across this manuscript.

Package	Application	Reference	Availability
**kuenm**	Principal component analysis (PCA), model calibration, selection, and calculation of evaluation metrics for *B. anthracis*.	[14]	https://github.com/manubio13/ku.enm
**raster**	Manipulation of raster files: crop, resample, mask, etc.	[15]	https://cran.r-project.org/web/packages/raster/vignettes/Raster.pdf
**SpThin**	Spatial thinning of *B. anthracis* occurrence records	[16]	https://cran.r-project.org/web/packages/spThin/spThin.pdf

S1 References

1. EMPRES-Animal health 360. FAO; 2022. doi:10.4060/cc2775en.
2. Mur L, Tizzani P, Awada L, Lambergeon N, Braham I, Masse C, et al. WAHIS, the unique source of official worldwide animal health information, is becoming OIE-WAHIS, a new digital platform. Front Vet Sci. 2019;6. doi:10.3389/conf.fvets.2019.05.00058.
3. European Commission. Animal Disease Information System (ADIS). 2023. Available: https://food.ec.europa.eu/animals/animal-diseases/animal-disease-information-system-adis_en.
4. Deka MA, Vieira AR, Bower WA. Modelling the ecological niche of naturally occurring anthrax at global and circumpolar extents using an ensemble modelling framework. Transbound Emerg Dis. 2022 [cited 19 Jul 2022]. doi:10.1111/TBED.14602.
5. Vega GC, Pertierra LR, Olalla-Tárraga MÁ. MERRAclim, a high-resolution global dataset of remotely sensed bioclimatic variables for ecological modelling. Sci Data 2017 41. 2017;4: 1–12. doi:10.1038/sdata.2017.78.
6. Wardlow BD, Egbert SL. A comparison of MODIS 250-m EVI and NDVI data for crop mapping: a case study for southwest Kansas. Httpsdoi-Orgareuabcat10108001431160902897858. 2010;31: 805–830. doi:10.1080/01431160902897858.
7. Hengl T, De Jesus JM, Heuvelink GBM, Gonzalez MR, Kilibarda M, Blagotić A, et al. SoilGrids250m: Global gridded soil information based on machine learning. PLOS ONE. 2017;12: e0169748. doi:10.1371/JOURNAL.PONE.0169748.
8. Poggio L, de Sousa LM, Batjes NH, Heuvelink GBM, Kempen B, Ribeiro E, et al. SoilGrids 2.0: producing soil information for the globe with quantified spatial uncertainty. SOIL. 2021;7: 217–240. doi:10.5194/soil-7-217-2021.
9. Gilbert M, Nicolas G, Cinardi G, Van Boeckel TP, Vanwambeke SO, Wint GRW, et al. Global distribution data for cattle, buffaloes, horses, sheep, goats, pigs, chickens and ducks in 2010. Sci Data 2018 51. 2018;5: 1–11. doi:10.1038/sdata.2018.227.
10. Gilbert M, Cinardi G, Da Re D, Wint WGR, Wisser D, Robinson TP. Global cattle distribution in 2015 (5 minutes of arc). Harv Dataverse. 2022. doi:10.7910/DVN/LHBICE.
11. Gilbert M, Cinardi G, Da Re D, Wint WGR, Wisser D, Robinson TP. Global goats distribution in 2015 (5 minutes of arc). Harv Dataverse. 2022. doi:10.7910/DVN/YYG6ET.
12. Gilbert M, Cinardi G, Da Re D, Wint WGR, Wisser D, Robinson TP. Global sheep distribution in 2015 (5 minutes of arc). Harv Dataverse. 2022. doi:10.7910/DVN/VZOYHM.
13. Booth TH. Checking bioclimatic variables that combine temperature and precipitation data before their use in species distribution models. Austral Ecol. 2022;47: 1506–1514. doi:10.1111/aec.13234.
14. Cobos ME, Townsend Peterson A, Barve N, Osorio-Olvera L. Kuenm: An R package for detailed development of ecological niche models using Maxent. PeerJ. 2019;2019: e6281. doi:10.7717/PEERJ.6281/SUPP-2.
15. Hijmans R, van Etten J. raster: Geographic analysis and modeling with raster data. R package version 2.0-12. 2012. Available: http://cran.r-project.org/package=raster
16. Aiello-Lammens ME, Boria RA, Radosavljevic A, Vilela B, Anderson RP. spThin: an R package for spatial thinning of species occurrence records for use in ecological niche models. Ecography. 2015;38: 541–545. doi:10.1111/ECOG.01132.
