# Supplementary material for: Suitability of anthrax (Bacillus anthracis) in the Black Sea basin through the scope of distribution modelling
Source: PLoS One. 2024 Nov 7;19(11):e0303413. doi: 10.1371/journal.pone.0303413 (PMC11542877; doi:10.1371/journal.pone.0303413)
Supplement: S2 File — (PDF) [file pone.0303413.s003.pdf]

Suitability of Anthrax (*Bacillus anthracis*) in the Black Sea Basin through the scope of distribution modelling

Margarida Arede, Alberto Allepuz, Daniel Beltran-Alcrudo, Jordi Casal, Daniel Romero-Alvarez

S2 File: Description of model outputs for non-selected models and Maxent output for selected approach.

- **Model outputs for non-selected approaches.**
- **S2 File Fig 1. Ecological niche modelling outputs for *Bacillus anthracis*.**
- **S2 File Table 1. Maxent output for approach 3.**
- **S2 References**

Model outputs for non-selected approaches

The two approaches based on variable combinations with only abiotic variables resulted in lower model performances. Model parameters and outputs for the two approaches that were not selected are presented in Table 1 (main text) and Figure 5, respectively.

The first approach used a PCA including all environmental variables. We kept the first five principal components (PCs) explaining 91.14% of the variance among all variables. This approach had a larger area predicted as suitable but a slightly higher omission rate and two additional parameters (Table 1, main text).

The second approach used PCAs in the environmental domain. We retained the first three PCs explaining 99.78% of the variance for humidity, 95.73% for soil, 98.87% for temperature, and one PC explaining 100% of the variance for vegetation greenness. The binary model (E=5%) for this approach (seen in S2 File Fig1, approach 2, panel C) yielded a smaller predicted area, using more than the double number of parameters compared with the other two approaches.

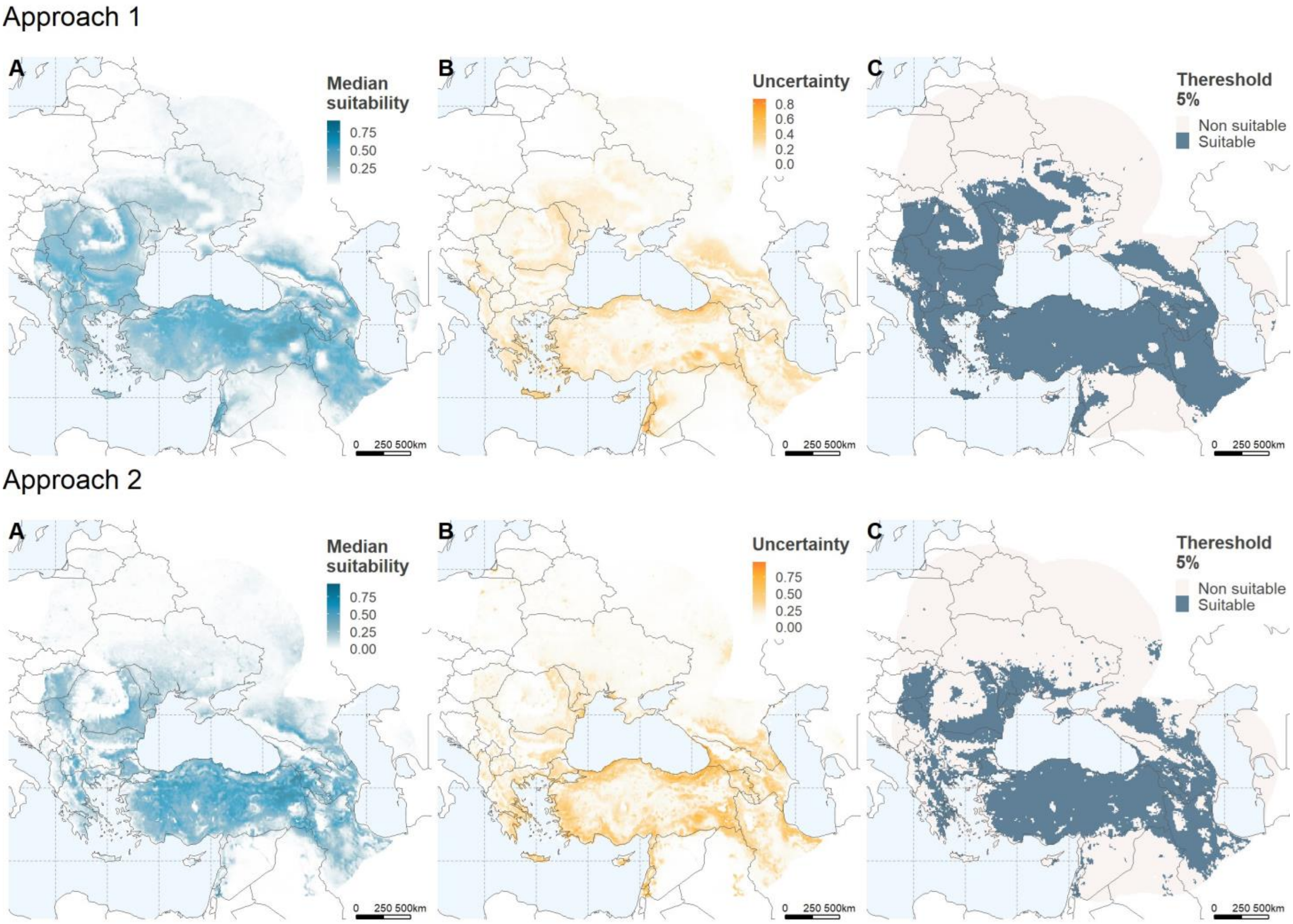

S2 File Fig 1. Ecological niche modelling outputs for *Bacillus anthracis*. Model outputs for *B. anthracis* using approach 1 (PCA for all selected variables) and approach 2 (PCA by domain). For both approaches, maps depict continuous suitability (A), uncertainty (B), and binary map of suitability using a 5% threshold (C). Maps were developed using shape files of *the world* from the public domain repository of Natural Earth (http://www.naturalearthdata.com/) and built using R Statistical Software (v4.2.1) [17].

S2 File Table 1. Maxent output for approach 3. Percent contribution and permutation importance (Maxent's automated output) for approach 3 (selected approach) in descending contribution order. The principal component (PC) three (PC3) from the temperature domain and the PC1 from the soil domain have the highest contribution for the selected model.

Variable	Percent contribution (%)	Permutation importance
Temperature PC 3	35.5	12
Soil PC 1	21.1	35.2
EVI PC 1	10.3	7.5
Ruminant abundance PC 1	9.9	1.1
Soil PC 2	6.5	16.8
Soil PC 3	5.3	13.6
Humidity PC 2	5	2.8
Humidity PC 1	3.7	1.8
Temperature PC 2	1.4	3.9
Temperature PC 1	1.3	5.2

S2 References

1. R Core Team. R: A language and environment for statistical computing. Vienna, Austria: R Foundation for Statistical Computing; 2023. Available: https://www.r-project.org/
